# Indigenous migration patterns in Brazil based on the 2010 national demographic census: analysis and critical reflection

**DOI:** 10.1007/s43545-021-00264-w

**Published:** 2021-10-06

**Authors:** Marden Barbosa de Campos, Ricardo Ventura Santos, Elaine Meire Vilela, Cláudia Lima Ayer de Noronha, Leandro Okamoto da Silva, Carlos E. A. Coimbra Jr, João Luiz Bastos, James R. Welch

**Affiliations:** 1 Faculdade de Filosofia e Ciências Humanas, Universidade Federal de Minas Gerais, Belo Horizonte, MG Brazil; 2 Escola Nacional de Saúde Pública, Fundação Oswaldo Cruz, Rio de Janeiro, RJ Brazil; 3 Instituto Brasileiro de Geografia E Estatística, Rio de Janeiro, RJ Brazil; 4 Departamento de Saúde Pública, Universidade Federal de Santa Catarina, Florianópolis, SC Brazil

**Keywords:** Human migration, Urbanization, Indigenous people, National censuses, Ethnic groups

## Abstract

Research in several Latin American countries points to violence, loss of traditional territories, and seeking education, health, and wage labor as key variables in triggering rural–urban migration among Indigenous people. This study presents an analysis of the migration patterns of Indigenous people in Brazil, compared to non-indigenous people, based on data from the most recent national census, conducted in 2010. Migration characteristics related to lifetime migration and recent migration were investigated by means of descriptive and multivariable logistic regression analyses. The findings pointed to complex mobility scenarios according to migrants’ Indigenous status and geographical regions of origin and destination. Indigenous people living in urban areas presented high levels of mobility (approximately 50% lived in different municipalities from those where they were born), which were more pronounced than those of non-Indigenous people. Indigenous people living in rural areas presented the lowest levels of migration (approximately 90% residing in their municipality of birth). Statistical modeling confirmed the patterns observed in descriptive analysis, highlighting the marked mobility of Indigenous subjects in urban areas. We emphasize the limitations of using census data for characterizing Indigenous mobility profiles, although no other nationally representative data are available. The finding that the Indigenous population living in urban areas presents rates of migration higher than their non-Indigenous counterparts is particularly important for the planning and implementation of a broad range of public policies aimed at ethnic minorities in the country, including health, education, and housing initiatives.

## Introduction

In international debates about Indigenous people and their relationships with nation states, the production of official statistics has been increasingly emphasized as a strategy to promote their visibility in national and global spheres (Axelsson and Sköld 2011; Anderson et al. 2016; Kukutai and Taylor 2016). The inclusion of Indigenous ethnic categories in population data banks is seen as a key factor in widening the scope of representation of different sociocultural groups in official statistics systems throughout the world (United Nations 2009). Statistical information has been considered strategically relevant for understanding the demographic history and dynamics of Indigenous people, largely influenced by colonization histories, with the potential to inform and expand public policies that recognize their sociocultural diversity and specificity.

In Latin America, especially since the 1990s, there has been a significant expansion in the collection of population data about Indigenous people, especially by means of national demographic censuses (Oliveira 1999; McSweeney and Arps 2005; Oyarce et al. 2009; Del Popolo et al. 2011; Angosto-Ferrández and Kradolfer 2012). As pointed out by Loveman (2014), many countries in the region have experienced major changes in governmental policies affecting ethnic minorities, including the recognition of Indigenous people, their legal rights, and need to be represented in national statistics. The production of demographic information for Indigenous populations in Latin America not only reflects a global trend toward enhancing statistical visibility of Indigenous minorities, but is also attributable to the strengthening of Indigenous political movements in the region (Oyarce et al. 2009; Angosto-Ferrández and Kradolfer 2012).

As in other Latin American countries, Brazil has also expanded data collection about Indigenous people in the national population census. According to the most recent census, conducted in 2010, the Indigenous population in Brazil totals 896 thousand individuals (0.4% of the general Brazilian population) (IBGE 2012). Although the Indigenous population in Brazil is proportionally small when compared to some other countries, there has been an increase in analyses aimed at understanding key demographic indicators of Indigenous populations such as mortality, fertility, spatial distribution, and access to public services, such as schooling and sanitation. These analyses have permitted documentation and dissemination of data evidencing the great divide that sets Indigenous people apart from their benchmark (non-Indigenous) Brazilian population (Perz et al. 2008; Santos et al. 2015, 2019a, b; Campos and Estanislau 2016; Bastos et al. 2017; Caldas et al. 2017; Pereira 2017).

Within the context of demographic analyses of Indigenous people in Brazil, investigations of their migration patterns have received relatively little attention, despite their centrality for understanding relationships between Indigenous people and Brazilian national society. Studies in other Latin American countries point out violence, loss of traditional territories, and seeking education, health, and wage labor as key variables in triggering rural–urban migration among Indigenous people (Del Popolo et al. 2011; Campbell 2015; McSweeney and Jokisch 2015; Wong and Sánchez 2014). With few exceptions (Estanislau 2014; Simoni and Dagnino 2016), investigations about Indigenous population migration in Brazil over the past decade have not relied on national census data, but instead use data from case studies aimed at understanding specific ethnic groups in local or regional contexts (Azevedo et al. 2013; Colman and Azevedo 2019; Teixeira et al. 2019). Macrolevel analyses about Indigenous migration patterns based on national statistics are scarce, thus limiting possibilities for intra and international comparisons.

The objective of this investigation is to analyze and critically reflect upon the migration patterns of Indigenous people in Brazil based on data from the most recent national census, conducted in 2010. Censuses are considered the most important source of quantitative information on migration produced in Brazil. The range of questions and the nationwide reach of the Census makes it the only database which permits understanding migration characteristics of the entire Brazilian population. An emphasis of the present study is on comparisons between migration in the Indigenous population in Brazil with non-Indigenous people, as well as between residents in rural and urban areas. After presenting our empirical analysis, we discuss the limitations of Brazilian census data for the purpose of characterizing Indigenous migration, particularly regarding rural–urban dynamics. Therefore, we develop a critical reflection, especially based on the anthropological literature addressing mobility patterns of Indigenous people in Brazil with a view to contributing to improvements in data collection on Indigenous migration within the scope of public statistics in Brazil.

## Methods

Analyses were based on data from the 2010 national demographic census, carried out by the Brazilian Institute of Geography and Statistics (IBGE 2013). The 2010 Census used two types of questionnaires: (1) a basic form with questions about household physical characteristics (e.g., rural or urban location, total household income, construction materials, and sanitation standard) and household residents (e.g., sex, age, literacy, and color or race classification following IBGE’s categories: “branco” [“white”], “preto” [“black”], “amarelo” [“yellow”], “pardo” [“brown”], and “indígena” [“Indigenous”]); (2) a sample questionnaire which, in addition to topics addressed in the basic questionnaire, included questions about household residents’ occupation, fertility, education, and migration, among other characteristics. The percentage of households in each municipality that were selected to respond to the sample questionnaire followed statistical procedures taking into consideration resident population size in each municipality, following standard census methodology, covering approximately 11% of the country’s households (IBGE 2013). For the purposes of this study, the color or race categories white, black, yellow, and brown were grouped into a single category, labeled “non-Indigenous.” In 2010, Brazil had 5565 municipalities, distributed in 26 states and one Federal District, all grouped into five geographical regions: North, Northeast, Southeast, South, and Central-West. The total population count in Brazil was 190,755,799 persons (84.6% urban; 15.6% rural), of which approximately 0.4% were classified as “Indigenous.”

The size of the Indigenous population calculated from the diverse databases released by the IBGE for the 2010 Census presents variations corresponding with the questionnaire format applied nationwide and within federally recognized Indigenous Lands. Officially, the Indigenous population in 2010 was 896,917 (IBGE 2012). This value includes a quantity of people declared Indigenous in response to the question regarding color or race (817,963 people, based on data collected using the “basic form”) as well as a quantity who did not declare Indigenous in response to this first question but did affirm that they “consider themselves Indigenous” in response to a second question applied only within officially recognized Indigenous Lands. This second question produced 78,954 affirmative responses.

Nevertheless, the databases released by the IBGE that include data related to migration only include data from the first question addressing color or race, not those pertaining to the other contingent, who responded to the second question applied within Indigenous Lands. In these databases with migration data, derived from the sample questionnaire, the total Indigenous population is 821,501 (slightly larger than the 817 thousand identified by means of the basic questionnaire). This small difference resulted from issues involving expansion of the sample. Therefore, for the purposes of the present investigation, the total Indigenous population is 821 thousand (39.2 urban and 60.8% rural).

The following migration data from the 2010 Census database were investigated: whether or not the person was born in the municipality of residence on the Census reference date, July 31, 2010 (lifetime migrants); the prior municipality of residence for people who resided for less than 10 years in the municipality of residence on the Census reference date, July 31, 2010 (place of last previous residence); and the municipality of residence on July 31, 2005 (fixed prior date) for those who resided for less than 6 years in the municipality of residence on the Census reference date, July 31, 2010 (IBGE 2013). The 2010 Census collected information about birth State (and, in the case of those not born in Brazil, birth country), but not about birth municipality or rural or urban situation of previous residence. Also, the 2010 Census methodology did not capture relocations of Indigenous and non-Indigenous subjects within the same municipality, such as those between rural and urban contexts, as migration events. The absence of collected data regarding previous rural or urban residence and internal migration within municipalities are important limitations for studies of Indigenous relocations based on census data. We return to this point in the Discussion, after presenting the study results.

For our analyses related to “fixed prior date,” although there is information about migrants’ prior municipality of residence, results are presented without indication of specific intermunicipal movements. This is because, after disaggregation of the total number of Indigenous migrants considering the more than 5 thousand municipalities in the country, the sample sizes for most municipalities are very reduced. Thus, this study uses multivariate models to investigate if the person migrated in the previous 10 years. In the case of analyses of “fixed prior date,” results are presented in aggregate according to geographical region, by means of a regional migration matrix of origin and destination.

Preliminary analyses indicated that whether people had migrated in the previous 10 years was largely structured by Indigenous status and urban/rural household location. The census data were therefore described according to a set of municipal- and individual-level characteristics for each of the four subsample groups indicated here: Indigenous people residing in either urban or rural areas, as well as non-Indigenous persons living in urban or rural areas. The municipal and individual variables according to which these four strata were described are listed in Table 1. Using logistic regression analyses, a statistical technique that has been previously employed in a number of migration studies (Schultz 1982; De Jong 2000; Curran and Rivero-Fuentes 2003), we estimated the impact of all independent variables (see Table 1) on the likelihood (i.e., probability) of having migrated in the previous 10 years (yes/no) within each of the four groups referred to above. In other words, the predicted probability of migration was calculated for each variable with all other covariates held at their means. In the few demographic case studies of Indigenous people’s migration in Brazil, several of these variables were identified as relevant to explain mobility patterns (Teixeira et al. 2019). Odds ratios and 95% confidence intervals were also estimated along with their statistical significance. The critical probability threshold was taken to be less than 5% (*p* < 0.05).

**Table 1:** Variables from the 2010 Brazilian national demographic census included in the descriptive and multivariable logistic regression analyses

Variable	Type	Category (1) and reference category (0)
Dependent variable		
Migrant	Dummy	1 = person migrated in the decade prior to the 2010 Census
		0 = person did not migrate in the decade prior to the 2010 Census
Independent variables		
Brazilian region		
North	Dummy	1 = North; 0 = Southeast
Northeast	Dummy	1 = Northeast; 0 = Southeast
South	Dummy	1 = South; 0 = Southeast
Central-West	Dummy	1 = Central-West; 0 = Southeast
Officially recognized indigenous lands in the municipality		
	Dummy	1 = yes; 0 = no
Municipal human development Index		
Low	Dummy	1 = 0.418–0.657; 0 = 0.740–0.862
Intermediate	Dummy	1 = 0.658–0.739; 0 = 0.740–0.862
Percent of whites in the municipality		
Low	Dummy	1 = 0.1%-31.6%; 0 = 54.7%-99.6%
Medium	Dummy	1 = 31.7%-54.6%; 0 = 54.7%-99.6%
Percentage of urban population in the municipality		
Low	Dummy	1 = 4.2%-69.1%; 0 = 94.9%
Medium	Dummy	1 = 69.2%-94.8%; 0 = 94.9%-100.0%
Household location	Dummy	1 = urban; 0 = rural
Indigenous status	Dummy	1 = Indigenous person; 0 = non-Indigenous person
Sex	Dummy	1 = male; 0 = female
Age group		
Age group 2	Dummy	1 = person in the 15–29 years group; 0 = person in the 0–14 years group
Age group 3	Dummy	1 = person in the 30–59 years group; 0 = person in the 0–14 years group
Age group 4	Dummy	1 = person in the 60 + year group; 0 = person in the 0–14 years group
Marital status	Dummy	1 = married or lived with a partner; 0 = single, divorced or widowed
Family income		
Income group 2	Dummy	1 = Between 1 and 2 minimum wages; 0 = Below 1 minimum wage
Income group 3	Dummy	1 = Between 2 and 5 minimum wages; 0 = Below 1 minimum wage
Income group 4	Dummy	1 = Above 5 minimum wages; 0 = Below 1 minimum wage
Education		
Education group 2	Dummy	1 = less than high school; 0 = undergraduate degree or higher
Education group 3	Dummy	1 = junior college or high school; 0 = undergraduate degree or higher
Education group 4	Dummy	1 = high school or undergraduate degree; 0 = undergraduate degree or higher

We also used logistic regression models to determine the municipal and sociodemographic profile of people with the highest and lowest probabilities of having migrated in the previous 10 years, among Indigenous subjects living in urban and rural areas. All analyses presented in this study are nationally representative, as they take into account the sampling weights and complex sampling design according to official IBGE recommendations.

## Results

The distribution of four groups according to municipal- and individual-level variables is presented in Table 2, which refers to the total number of Indigenous and non-Indigenous people in Brazil in 2010. We address the Indigenous population, as per the focus of the study explained above. This table shows that Indigenous people residing in rural areas were more likely to live in the Northern and Northeastern regions of the country. Higher percentages of Indigenous people living in urban areas were observed in the Northeast and the Southeast, on the other hand. Table 2 also indicates that Indigenous people living in rural areas are highly concentrated (~ 95%) in municipalities containing officially recognized Indigenous Lands. Non-Indigenous subjects living in urban areas were more likely to reside in municipalities with intermediate and high levels of Human Development Index, higher percentages of people who declared their color or race as white, and higher proportions of urban population. The age structure was fairly similar across the groups considered, except for Indigenous people residing in rural areas, who showed an expressively younger age profile. Indigenous subjects living in rural areas were not only the poorest among all groups analyzed, but also showed the lowest levels of education.

**Table 2:** Description of subsample groups according to municipal- and individual-level characteristics (migrants and non-migrants), 2010 Brazilian national demographic census

Subject characteristics	Indigenous*		Non-indigenous	
	Rural	Urban	Rural	Urban
Migrated in the previous 10 years				
No	95.0	81.7	86.7	84.6
Yes	5.0	18.3	13.3	15.4
Brazilian region				
North	50.9	19.4	14.3	7.7
Northeast	20.0	34.4	50.2	24.5
Southeast	4.1	29.0	20.4	49.3
South	5.4	7.1	9.8	10.2
Central-West	19.7	10.1	5.4	8.2
Officially recognized indigenous lands in the municipality				
No	5.8	66.3	80.5	85.7
Yes	94.2	33.7	19.5	14.3
Municipal human development index				
Low (0.418–0.657)	79.6	31.2	65.2	15.3
Intermediate (0.658–0.739)	16.1	27.7	26.1	30.0
High (0.740–0.862)	4.3	41.2	8.7	54.7
Percentage of whites in the municipality				
Low (0.1–31.6%)	77.7	47.1	54.0	24.8
Medium (31.7–54.6%)	15.6	31.6	26.7	38.0
High (54.7–99.6%)	6.7	21.2	19.3	37.2
Percentage of urban population in the municipality				
Low (4.2–69.1%)	79.4	24.2	68.5	12.2
Medium (69.2–94.8%)	19.3	28.5	27.4	28.8
High (94.9–100.0%)	1.3	47.3	4.1	59.0
Age groups (years)				
60 +	5.8	10.9	10.7	10.6
30–59	21.4	39.3	34.2	38.9
15–29	27.3	27.7	26.3	27.0
0–14	45.5	22.1	28.8	23.4
Marital status				
Married or with a partner	65.4	57.0	56.7	57.8
Single, divorced or widowed	34.6	43.0	43.3	42.2
Family income (minimum wages)				
5 +	0.3	2.7	1.2	5.8
2–5	0.3	7.9	2.9	13.5
1–2	2.5	22.0	12.0	25.3
< 1	96.9	67.4	84.0	55.4
Education				
Less than high school	89.3	61.6	80.2	54.1
Junior college or high school	7.0	14.9	11.1	15.4
High school or undergraduate degree	3.3	19.4	7.6	22.3
Undergraduate degree or higher	0.4	4.2	1.0	8.2
Sex				
Male	48.5	52.8	47.2	51.7
Female	51.5	47.2	52.8	48.3

Of the total of Indigenous people counted in the 2010 Census, 26.2% lived in municipalities distinct from their birthplaces. There are important contrasts when results are stratified according to rural and urban location (10.2% and 51.0%, respectively). For non-Indigenous people, the percentage of lifetime migrants was 30.0% in 2010 (43.5% for those living in urban areas and 30.0% living in rural areas). Therefore, considering lifetime migrants, whereas the total Indigenous population migrated less than the non-Indigenous population, a much higher proportion of migrants was observed among Indigenous people residing in urban areas (51.0%), while the lowest frequency was documented in rural areas (10.2%), constituting distribution extremes.

Comparing 2005 and 2010, analyses show different migration patterns between municipalities for the Indigenous and non-Indigenous populations in the five geographical regions (Table 3). Because the 2010 Census did not collect information about whether migrants’ prior residences were in rural or urban areas, these migratory flows refer to both contexts combined. Of the total of Indigenous people who migrated between municipalities during the period, the largest outflows occurred in municipalities in the Northeast (34.3%) and Southeast (22.8%) regions, which were also those which received more Indigenous migrants (24.9% e 31.5%, respectively). These results, which point to more expressive inflows and outflows in the same two regions, involve migration between municipalities in the same region (principal diagonals) and between municipalities in different regions. Considering only flows between regions, the Northeast stands out as the region from which proportionally more Indigenous people left (34.3–11.0%, or 23.3%) and the Southeast as that which received more (31.5–7.3%, or 24.2%). Specifically, the principal migratory flow of Indigenous people was between municipalities in the Northeast toward the Southeast (16.0%). In general terms, these patterns described for Indigenous people are similar to those observed among non-Indigenous people. However, there are differences. For example, the importance of the North region as the origin of Indigenous migrants (17.4%) was consistently larger when compared to non-Indigenous migrants (9.1%), especially among those who moved to municipalities in the Central-West (5.9%). Another aspect to highlight is that the proportions of intraregional migrations (represented in the principal diagonals of Table 3) are slightly more elevated for Indigenous people for almost all regions, with the exception of the Southeast, indicating greater occurrence of short distance moves (between closely located municipalities) than occurred in the non-Indigenous population.

**Table 3:** Regional migration matrix depicting frequencies of individuals by geographical region of residence in 2005 and 2010, Brazilian national demographic census

Place of residence (on July 31, 2005)	Place of residence (on July 31, 2010)					
	North	Northeast	Southeast	South	Central-West	Total
Indigenous						
North	5.7	2.2	2.8	0.8	5.9	17.4
Northeast	1.7	11.0	16.0	1.1	4.4	34.3
Southeast	0.5	9.8	7.3	2.5	2.8	22.8
South	0.0	0.4	2.7	8.7	0.7	12.6
Central-West	1.4	1.5	2.8	2.3	4.9	12.9
Total	9.4	24.9	31.5	15.4	18.7	
Non-Indigenous						
North	3.5	1.7	1.2	0.5	2.2	9.1
Northeast	3.2	7.9	17.8	1.1	5.3	35.3
Southeast	1.2	8.3	13.1	4.5	4.0	31.1
South	0.5	0.6	3.3	7.1	1.5	12.9
Central-West	1.6	1.7	2.8	1.3	4.2	11.5
Total	9.9	20.2	38.1	14.6	17.2	

Results of multivariable regression analysis (Table 4) point to differentials in the probabilities of migration between Indigenous and non-Indigenous people, according to rural or urban household location in 2010. Likelihood and odds ratio results present similar patterns overall. In alignment with descriptive analyses detailed above for the lifetime migration results, Indigenous persons residing in urban areas showed the greatest likelihood of migrating among all groups examined, while Indigenous persons living in rural areas had the lowest probabilities of migrating in the previous 10 years. Table 4 also shows that Indigenous and non-Indigenous individuals living in some Brazilian regions (Central-West, South, and North) had a higher likelihood to have migrated in recent years (both within municipalities in the region or coming from other regions), while the other municipal-level variables had a less pronounced impact on migration probabilities. As for the individual-level variables, regression models revealed that the two youngest age groups (0–14 and 15–29 years) showed the highest probabilities of migration among Indigenous and non-Indigenous persons. While marital status and level of education had a modest impact on migration probabilities, family income had a greater influence on this dependent variable: the higher the income, the higher the probability of people having migrated in the previous 10 years.

**Table 4:** Likelihood (predicted probability with all other covariates held at their means) and odds ratios of having migrated in the previous 10 years among Indigenous and non-Indigenous subjects living in urban and rural areas, according to covariate categories, 2010 Brazilian national demographic census

Covariate	Indigenous subjects				Non-indigenous subjects			
	Residing in rural areas		Residing in urban areas		Residing in rural areas		Residing in urban areas	
	Likelihood	OR (CI95%)	Likelihood	OR (CI95%)	Likelihood	OR (CI95%)	Likelihood	OR (CI95%)
Unweighted sample size/weighted sample size	75,384/ 483,604		31,226/ 306,344		4,358,469/ 27,520,594		14,680,735/ 150,547,781	
Brazilian region								
North	5.2	1.0	20.9	1.0	15.1	1.0	16.9	1.0
Northeast	3.7	2.4 (2.0; 2.9)*	15.7	0.9 (0.8; 1.1)	11.1	0.6 (0.6; 0.6)*	12.5	0.8 (0.8; 0.8)*
Southeast	4.1	3.5 (2.6; 4.7)*	17.1	1.2 (1.0; 1.5)	12.2	0.8 (0.8; 0.8)*	13.7	0.8 (0.8; 0.9)*
South	5.6	3.1 (2.0; 4.6)*	22.1	1.1 (0.8; 1.5)	16.0	0.7 (0.6; 0.7)*	17.9	1.1 (1.1; 1.1)*
Central-West	7.3	2.4 (1.8; 3.2)*	27.5	1.5 (1.2; 1.9)*	20.3	1.7 (1.6; 1.7)*	22.6	1.4 (1.4; 1.4)*
Officially recognized Indigenous Lands in the municipality								
No	4.5	1.0	18.4	1.0	13.2	1.0	14.8	1.0
Yes	4.0	0.2 (0.2; 0.3)*	16.7	0.7 (0.6; 0.8)*	11.9	1.0 (1.0; 1.0)	13.4	0.8 (0.8; 0.8)
Municipal human development index								
Low (0.418–0.657)	3.9	1.0	16.3	1.0	11.6	1.0	13.0	1.0
Intermediate (0.658–0.739)	5.3	1.2 (1.0; 1.5)	21.3	1.2 (1.0; 1.5)	15.4	1.4 (1.4; 1.5)*	17.3	1.1 (1.1; 1.1)*
High (0.740–0.862)	4.2	0.9 (0.6; 1.4)	17.3	0.8 (0.7; 1.0)	12.3	1.4 (1.3; 1.4)*	13.9	0.9 (0.9; 0.9)*
Percent of whites in the municipality								
Low (0.1%-31.6%)	4.3	1.0	17.7	1.0	12.6	1.0	14.2	1.0
Medium (31.7%-54.6%)	4.4	0.9 (0.7; 1.1)	18.2	1.2 (1.0; 1.3)*	13.0	1.0 (1.0; 1.0)	14.6	1.0 (1.0; 1.0)*
High (54.7%-99.6%)	4.5	1.9 (1.3; 2.8)*	18.5	1.8 (1.5; 2.2)*	13.2	1.1 (1.1; 1.2)*	14.8	1.0 (1.0; 1.0)*
Percentage of urban population in the municipality								
Low (4.2%-69.1%)	4.1	1.0	17.1	1.0	12.2	1.0	13.7	1.0
Medium (69.2%-94.8%)	5.0	1.0 (0.8; 1.2)	20.4	1.1 (0.9; 1.4)	14.7	1.0 (1.0; 1.0)	16.5	1.0 (1.0; 1.1)*
High (94.9%-100.0%)	4.2	2.7 (1.6; 4.8)*	17.4	1.1 (0.9; 1.4)	12.4	0.9 (0.8; 0.9)*	14.0	0.9 (0.9; 0.9)*
Age groups (years)								
60 +	2.3	1.0	10.1	1.0	7.0	1.0	7.9	1.0
30–59	4.2	1.3 (1.1; 1.6)*	17.4	2.1 (1.8; 2.4)*	12.4	2.0 (1.9; 2.0)*	14.0	1.8 (1.8; 1.8)*
15–29	5.7	1.7 (1.3; 2.1)*	22.6	4.1 (3.5; 4.8)*	16.4	2.8 (2.7; 2.8)*	18.3	3.0 (3.0; 3.1)*
0–14	4.8	1.5 (1.2; 1.9)*	19.4	3.4 (2.9; 4.0)*	13.9	3.5 (3.5; 3.6)*	15.6	2.8 (2.7; 2.8)*
Marital status								
Married or with a partner	4.2	1.0	17.4	1.0	12.4	1.0	14.0	1.0
Single, divorced or widowed	4.7	1.3 (1.2; 1.5)*	19.2	1.4 (1.3; 1.6)*	13.8	1.6 (1.6; 1.6)*	15.5	1.4 (1.4; 1.4)*
Family income (minimum wages)								
5 +	6.4	1.0	24.9	1.0	18.2	1.0	20.3	1.0
2–5	4.9	1.1 (0.5; 2.1)	19.8	0.7 (0.6; 1.0)*	14.2	0.3 (0.3; 0.3)*	16.0	0.7 (0.7; 0.7)*
1–2	4.3	0.8 (0.5; 1.3)	17.8	0.6 (0.5; 0.8)*	12.7	0.3 (0.2; 0.3)*	14.3	0.6 (0.6; 0.6)*
< 1	4.2	0.5 (0.3; 0.8)*	17.5	0.6 (0.4; 0.8)*	12.5	0.2 (0.2; 0.2)*	14.0	0.6 (0.6; 0.6)*
Education								
Less than high school	4.2	1.0	17.3	1.0	12.3	1.0	13.8	1.0
Junior college or high school	4.5	1.2 (1.0; 1.4)*	18.6	1.0 (0.9; 1.2)	13.3	1.1 (1.1; 1.1)*	14.9	0.9 (0.9; 0.9)*
High school or undergraduate degree	4.8	1.1 (0.9; 1.4)	19.4	1.0 (0.9; 1.2)	13.9	1.1 (1.1; 1.1)*	15.6	1.0 (1.0; 1.0)*
Undergraduate degree or higher	5.4	1.2 (0.7; 2.0)	21.5	1.5 (1.2; 1.8)*	15.6	1.2 (1.1; 1.2)*	17.4	1.1 (1.1; 1.1)*
Sex								
Female	4.5	1.0	18.5	1.0	13.2	1.0	14.8	1.0
Male	4.3	1.0 (0.9; 1.1)	17.9	1.0 (0.9; 1.1)	12.7	1.0 (1.0; 1.0)	14.3	1.0 (1.0; 1.0)*

*OR* odds ratio, *CI* confidence interval

*Statistically significant at 5%

Following logistic regression analysis (results not shown in tables), Indigenous subjects living in urban areas with the highest probability of migration (i.e., 60.2%) had the following profile: their preferred destinations were municipalities in the Central-West, they moved into municipalities where there were officially recognized Indigenous lands, whose Human Development Index was intermediate (0.658–0.739), and where there was a high (54.7–99.6%) proportion of whites, as well as medium levels (69.2–94.8%) of urban population. These migrants were between 15 and 29 years of age, single, divorced or widowed, had family incomes equal to or higher than 5 minimum wages, and high levels of education (undergraduate degree or higher). The profile of Indigenous people residing in rural areas with the lowest likelihood of migration (i.e., 1.2%) was as follows: their preferred destinations were municipalities in the Northeast, where there were officially recognized Indigenous lands, and which showed low Human Development Index (0.418–0.657), low proportion of whites (0.1–31.6%), and low frequency of urban population (4.2–69.1%). These subjects were also 60 years of age and over, married or living with a partner, and had low levels of family income (< 1 minimum wages) and education (less than high school).

## Discussion

The findings of this study, which focuses on migration patterns of the Indigenous population in Brazil according to data from the 2010 National Census, point to complex mobility scenarios, especially but not only as they relate to the urban/rural dimension. An important finding is what might be called a polarization of migration profiles in the case of Indigenous people when comparing place of birth and place of residence in 2010. While Indigenous people living in urban areas presented high levels of lifetime mobility (approximately 50% lived in municipalities in which they were not born), which were more pronounced than for non-Indigenous people, those living in rural areas presented the lowest levels (approximately 90% resided in their municipality of birth). Also, when comparing geographical regions of residence in 2005 and 2010, it was observed that the major migratory flow for Indigenous people was from the Northeast toward the Southeast, a pattern also observed in the non-Indigenous population. Furthermore, 2010 Census data showed greater occurrence of short distance moves in the form of migration between municipalities in the same geographical region among Indigenous people as compared to non-Indigenous people. Statistical modeling confirmed patterns observed in the descriptive analysis, highlighting the marked mobility of Indigenous subjects who resided in urban areas in 2010. For Indigenous subjects in urban areas, the variables age (0–14 and 15–29 years) and income were associated with higher probability of having migrated in the prior ten years.

To analyze Indigenous people’s demography in Brazil, including migration patterns derived from Brazilian census data, a series of specificities should be taken into consideration. Of all Latin American countries, Brazil has one of the smallest relative Indigenous populations, which comprises less than 0.5% of the total population (IBGE 2012; Santos et al. 2019a, b). Despite its small relative size, the Indigenous population in Brazil has enormous ethnic and linguistic diversity. With more than 300 Indigenous ethnic groups and over 200 distinct Indigenous languages, Brazil is one of the countries with the greatest Indigenous ethnic diversity in the world (IBGE 2012; Coimbra et al. 2013; Santos et al. 2019a, b). Furthermore, in contrast to many other Latin American countries, from the early twentieth century to the early 2010s, the Brazilian government implemented and administered policies for identification and demarcation of lands that improved Indigenous people’ usufruct of traditional territories (Lima 2005; Oliveira 2018). As a result, currently about 13% of the Brazilian territory is demarcated as federal Indigenous lands (IBGE 2012).

While recognizing the centrality of generally beneficial land policies directed toward Indigenous people in Brazil from the last century until recently, there remain problems and tensions in many regions of the country. Particularly in the Northeast, Southeast, and South, the areas of earliest colonization, Indigenous lands tend to be smallest and most urbanized, densely populated, limited in their subsistence production potentials, and restricted in their residential capacities (Oliveira 2011). Recent decades have been marked by a country-wide escalation of agrarian conflicts and violence involving Indigenous territories, including invasions of recognized Indigenous lands by non-Indigenous squatters and economic, demographic, and political pressure (including from agribusiness interests and the agricultural lobby) for the non-recognition of new Indigenous lands (Sullivan 2013; Carneiro da Cunha et al. 2017). Small, overpopulated lands and conflicts with squatters, miners, agribusiness, and other non-Indigenous interests may be among the driving forces of rural–urban migration.

This study analyzed migration data for the Indigenous population from the 2010 Brazilian Census from a critical perspective regarding the potential findings and interpretations derived from a survey not originally designed to attend to the specificities of this specific population segment. Although this data source is the only one in the country that potentially supports nationally representative analyses of population migration, the 2010 Census data present limitations with regard to characterizing the diverse nuances of Indigenous people’ mobility. The absence of data on intra-municipality migration and urban/rural condition of migration origin and destination locations directly affects analyses of the Indigenous population. Neither do there exist census data regarding migration according to Indigenous ethnic group or whether a person lived in an Indigenous land, which is in part due to limitations of data disaggregation considering these data were collected by means of Census sample methodology and the population segment of concern is relatively small.

Additionally, Census analytical categories generate potential comprehension difficulties when applied by means of interviews to culturally and often linguistically differentiated segments of the population. For example, geographical designations such as municipality and state, which are fundamental to the group of Census questions about migration, may not be familiar to some Indigenous people with distinct lexicons of geographical and spatial classification. Also, census data collection instruments, structured for the national non-Indigenous population, include socioeconomic and cultural categories, such as income, employment, and co-residence with a conjugal partner, that may not be adequate to characterize the realities experienced by culturally differentiated populations. As has been noted in diverse historical and socio-anthropological analyses, many surveys, such as censuses, are primordially structured to capture data about “average citizens” (see Igo 2007; Campos and Estanislau 2016).

Therefore, our understanding and intent are that the analyses presented in this study should not be interpreted as constituting a nuanced characterization of migration profiles for the historically and ethnically plural Indigenous people in Brazil, but rather as an attempt to explore the potentialities as well as the limitations of available census data. Even given these limitations, the analyses reveal diverse aspects of the Indigenous population’s migration profiles that strike us as consistent with historical and contemporary contexts in Brazil.

Results of the present investigation indicate that mobility levels were lowest for Indigenous residents of rural areas in comparison to Indigenous urban and non-Indigenous rural and urban residents. Furthermore, the great majority of Indigenous residents in rural areas in 2010 lived in their municipalities of birth. Migration indicators in years immediately before 2010 also reflect this pattern, as did results of multivariate analysis, which took into consideration the existence of possible differences in the population composition (by age, schooling, distribution by region, among other variables). Based on these findings, we believe it is reasonable to argue that the historical existence of federal public policies aimed at recognizing Indigenous lands, although quite insufficient to meet the most reasonable of demands of the Indigenous movement, have a role in explaining this scenario of low mobility of the Indigenous population residing in the country’s rural areas. Most of the self-identified Indigenous population in rural areas resided in municipalities containing Indigenous lands (in 2000, 86.7% of the Indigenous population residing in rural areas lived in municipalities containing Indigenous lands) or lived in Indigenous lands (in 2010, 85.9% of the rural Indigenous population resided on Indigenous lands) (IBGE 2005, 2012).

This scenario of low mobility should be contextualized considering the temporal scale encompassed by the census data. There are rich and diverse historical and anthropological texts which point out that many Indigenous people were markedly mobile in the past, with movements and migrations being associated with subsistence and economic activities, warfare, and rituals, among others (Hemming 1987; Roller 2014; Alexiades 2015). National census data, including those derived from 2010 Census, inform only about the migration profile of interviewees relative to birthplace and up to ten years prior to the Census reference date, which is a somewhat restricted temporal dimension. Therefore, care should be taken in interpreting Census data indicating low migration rates for Indigenous residents of rural areas in Brazil, because one cannot generalize beyond the specific timeframes of the Census, especially about Indigenous people’ long and complex colonization experiences.

Results addressing Indigenous residents of urban areas point to a markedly different scenario than was observed for the rural population, as a significant portion of Indigenous residents of urban areas have migrated at least once during their lifetimes. Therefore, the census data suggest the issue of Indigenous migration in contemporary Brazil is intrinsically related to urban contexts. Just as we mentioned above that the recognition of Indigenous lands may be a relevant factor for interpreting low migration levels of residents of rural areas, the tense land situation in many areas of the country may similarly be related to the high mobility observed in this study among Indigenous people residing in urban contexts. This probability derives from the likelihood that some of the Indigenous migrants residing in urban areas in 2010 may have moved during their lifetimes or in recent years from rural areas.

For several decades, and especially since 2000, the issue of the Indigenous population’s urbanization in Brazil has been the subject of in-depth anthropological investigation (Cardoso de Oliveira 1968; Andrello 2006; Fígoli and Fazito 2009; Magnani and Andrade 2013; Roller 2014; Azevedo et al. 2013; Garcés 2014; Alexíades and Peluso 2015). These studies, generally addressing specific ethnic groups, have explored processes underlying the mobility of Indigenous people to urban areas, revealing a wide and intricate range of historically and socially specific processes. Although this ethnographic literature suggests the importance of caution when seeking universalizing explanations for rural–urban migration nationally, it also points to several trends that apply to multiple ethnic groups throughout the country. For example, anthropologists have emphasized such migration triggers as the loss of traditional territories through invasion or infrastructure projects (e.g., dams and highways), violence associated with territorial conflict, as well as the desire to live in proximity to schools, health services, and employment opportunities (Cardoso de Oliveira 1968; Ferri 1990; Bernal 2009; Nakashima and Albuquerque 2011; Andrade et al. 2013).

It is not possible to establish specific causes of migration based on Census data (IBGE 2013). Nevertheless, our multivariate analyses show associations between potential explanatory variables (age and income) and migration of Indigenous people residing in urban areas in 2010. Notwithstanding, in interpreting the similarity observed between explanatory variables for Indigenous and non-Indigenous migration, it should be remembered that the Census variables available for analysis and utilized in this study, including those related to multi-level characteristics, may be too limited to portray the complex determinants of migration patterns among Indigenous residents in urban areas. As already pointed out, neither for descriptive nor for multivariate analyses was information available regarding whether migrants came from urban or rural areas, causing the aggregation of what are likely to be intrinsically heterogeneous populations. Also, given that the census data did not capture urban–rural migration within the same municipality, a potentially significant contingent of short distance moves, very common among the Indigenous population, are not represented in the census data. Diverse anthropological studies have highlighted that short- and long-distance mobility of Indigenous people who move from Indigenous lands to regional urban centers is a common phenomenon in various parts of Brazil, including Amazonia (Campbell 2015; Peluso 2015; Santos et al. 2019a, b).

In conclusion, the findings of this study reveal a complex configuration of socioeconomic factors associated with the migration dynamics of the Indigenous segment of the Brazilian population. The finding that the Indigenous population living in urban areas presents rates of migration higher than their non-Indigenous counterparts is particularly important for the planning and implementation of a broad range of public policies aimed at ethnic minorities in the country, including health, education, and housing initiatives. We also emphasize the limitations of using census data for characterizing Indigenous mobility profiles, although no other nationally representative data are available. Future efforts should aim to advance knowledge of the migration patterns of the Indigenous population by means of in-depth studies of local and regional contexts based on Census and other data. Also, the range of variables collected by Brazilian national statistics about Indigenous people could be broadened. This could take place through various means, including post-enumeration national and or regional studies, which should aim at characterizing their population features in a more nuanced way, including the use of more social-economic and culturally sensitive categories.

## Data Availability

Data are publicly available from the Brazilian Institute of Geography and Statistics (IBGE) at the website https://downloads.ibge.gov.br/.

## Declarations

### Code availability

Analyses were conducted using Stata 12.
